# Efficacy and safety of Compound Danshen Dripping Pills as an adjunct to conventional therapy for stable angina pectoris: an updated systematic review and meta-analysis of randomized controlled trials

**DOI:** 10.3389/fphar.2026.1952286

**Published:** 2026-09-24

**Authors:** Xiaoyue Zhao, Yaoyuan Liu, Liying Yang, Zeqi Chen, Wenjun Zhang, Weijing Lu, Yingda Zhao, Zhicheng Zhou, Fengwen Yang, Wentai Pang

**Affiliations:** 1 Evidence-Based Medicine Center, Tianjin University of Traditional Chinese Medicine, Tianjin, China; 2 College of Traditional Chinese Medicine, Tianjin University of Traditional Chinese Medicine, Tianjin, China; 3 College of Acupuncture and Tuina, Tianjin University of Traditional Chinese Medicine, Tianjin, China

**Keywords:** Compound Danshen Dripping Pills, coronary artery disease, meta-analysis, randomized controlled trials, stable angina pectoris, systematic review

## Abstract

**Objective:**

To systematically evaluate the efficacy and safety of Compound Danshen Dripping Pills (CDP) in the treatment of stable angina pectoris (SAP) and provide updated evidence regarding its clinical application.

**Methods:**

A comprehensive literature search was conducted in eight electronic databases, including PubMed, Embase, Web of Science, Cochrane Library, CNKI, Wanfang Data, VIP, and SinoMed, from inception to 31 May 2026. Randomized controlled trials (RCTs) comparing CDP plus conventional therapy (CT) with CT alone or CT plus nitrates in patients with SAP were included. Primary outcomes included angina efficacy, electrocardiogram (ECG) efficacy, angina frequency, angina duration, and nitroglycerin consumption. Secondary outcomes included lipid profile, left ventricular ejection fraction (LVEF), and fibrinogen (FIB), while adverse events were assessed as safety outcomes. Meta-analysis was performed using Review Manager 5.4 and Stata 18.0, and the certainty of evidence was evaluated using the GRADE framework.

**Results:**

A total of 93 RCTs involving 10,208 participants were included. Compared with CT alone, adjunctive CDP was associated with improvements in angina efficacy (RR = 1.26, 95% CI: 1.19–1.33), ECG efficacy (RR = 1.34, 95% CI: 1.19–1.51), reduced angina frequency (SMD = −1.32, 95% CI: −1.79 to −0.84) and duration (SMD = −2.51, 95% CI: -3.58 to −1.45), and increased LVEF (SMD = 1.30, 95% CI: 0.24–2.37). Compared with nitrates plus CT, CDP plus CT also demonstrated comparable or superior improvements in angina efficacy (RR = 1.11, 95% CI: 1.08–1.15), ECG efficacy (RR = 1.35, 95% CI: 1.30–1.40), angina frequency, lipid profile, FIB, and several functional outcomes. CDP was generally well tolerated, with no increased risk of adverse events compared with CT alone and a lower incidence than nitrate-based therapy. However, the certainty of evidence ranged from moderate to very low, mainly due to methodological limitations and inconsistency among included studies.

**Conclusion:**

Current evidence suggests that CDP-containing regimens may improve symptomatic and functional outcomes in patients with SAP compared with CT alone or nitrate-containing regimens; however, the certainty of evidence remains limited, and well-designed RCTs are needed to confirm these findings.

**Systematic Review Registration:**

https://www.crd.york.ac.uk/PROSPERO/myprospero, Identifier CRD420261416404.

## 1 Introduction

Stable angina pectoris (SAP), primarily caused by obstructive coronary artery disease (CAD), is a clinical syndrome characterized by transient myocardial ischemia resulting from an imbalance between myocardial oxygen supply and demand (Chinese Society of Cardiology, Chinese Medical Association, 2024). As one of the major manifestations of ischemic heart disease (IHD), SAP contributes substantially to the global burden of cardiovascular disease. In the United States, chronic coronary disease affects more than 20 million individuals, including over 10 million patients with stable angina (Virani et al., 2023), while in China the prevalence of coronary heart disease has exceeded 11 million cases (National Center for Cardiovascular Diseases, and Writing Group of the Report on Cardiovascular Health and Diseases in China, 2025). Population aging and the increasing prevalence of metabolic disorders, such as diabetes and obesity, are expected to further increase the burden of chronic stable angina, underscoring the need for additional therapeutic strategies to improve symptom control and long-term outcomes.

Pharmacological management for SAP primarily aims to relieve symptoms, improve quality of life, and reduce the risk of major adverse cardiovascular events (MACE). Current guideline-recommended therapies include prognostic medications, such as antiplatelet agents, lipid-lowering drugs, beta-blockers, and renin-angiotensin-aldosterone system (RAAS) inhibitors, together with symptomatic treatments including nitrates and calcium channel blockers (CCBs) (Chinese Society of Cardiology, Chinese Medical Association, 2024). However, despite advances in pharmacological management, some patients continue to experience persistent symptoms or treatment-related adverse effects, indicating an unmet clinical need for complementary therapeutic approaches. Traditional Chinese Medicine (TCM) has been widely applied in the management of SAP and has demonstrated potential benefits in alleviating angina symptoms, reducing myocardial ischemia, and improving quality of life, thereby providing a complementary therapeutic approach.

Compound Danshen Dripping Pills (CDP) is a commercial Chinese polyherbal preparation (CCPP) widely used for coronary artery disease and stable angina pectoris in China. According to the Chinese Pharmacopoeia (Chinese Pharmacopoeia Commission, 2025), the reference CDP formulation contains *Salvia miltiorrhiza* Bunge [Lamiaceae; Salviae miltiorrhizae radix et rhizoma], *Panax notoginseng* (Burkill) F.H.Chen [Araliaceae; Notoginseng radix et rhizoma], and synthetic borneol (Borneolum Syntheticum) at a starting-material ratio of 90:17.6:1. Taxonomic validation details are provided in Appendix A. The botanical drugs undergo water extraction and ethanol precipitation, and the resulting extract is combined with synthetic borneol and polyethylene glycol to form dripping pills. Pharmacopoeial quality control includes chromatographic identification and a minimum danshensu content of 0.10 mg per pill. CDP is traditionally used to promote blood circulation and relieve pain, while modern pharmacological studies suggest that it may improve myocardial ischemia, inhibit platelet aggregation, protect vascular endothelial function, enhance microcirculation, and alleviate coronary vasospasm. These effects may contribute to improved coronary blood flow and myocardial perfusion (Writing Group for the Expert Consensus on the Clinical Application of Compound Danshen Dripping Pills, 2025). Recent studies have further highlighted the cardiovascular protective potential of natural products through regulation of vascular function and oxidative stress-related pathways (Sun et al., 2026). Clinical evidence suggests that CDP may reduce angina symptoms and improve quality of life in patients with SAP, supporting its potential as an adjunctive therapeutic option.

To date, CDP has been recommended by several Chinese expert consensus statements for the management of SAP (Writing Group for the Expert Consensus on the Clinical Application of Compound Danshen Dripping Pills, 2025). Several systematic reviews and meta-analyses have evaluated CDP for angina pectoris, with substantial differences in populations, comparators, outcomes, and methodological approaches. Earlier reviews predominantly compared CDP with isosorbide dinitrate, whereas more recent syntheses have evaluated CDP versus nitrates in SAP and CDP combined with isosorbide mononitrate (Li et al., 2011; Jia et al., 2012; Jia and Leung, 2017; Zhang et al., 2023; Wang et al., 2024). Nevertheless, previous efficacy-focused reviews did not systematically determine whether the identity, composition, preparation, product specifications, phytochemical characterization, and quality control of the investigated CDP preparations were reported sufficiently to support cross-trial comparability and reproducibility. The present review therefore updates the randomized evidence within two predefined background-therapy frameworks and applies the ConPhyMP framework to evaluate preparation-specific reporting, while using RoB 2 and GRADE to reassess the reliability and certainty of the available evidence.

## 2 Materials and methods

### 2.1 Study registration

The protocol of this systematic review was registered in PROSPERO (CRD420261416404), and the study was conducted in accordance with the Preferred Reporting Items for Systematic Reviews and Meta-Analyses (PRISMA 2020) statement (Page et al., 2021).

### 2.2 Ethical statement

As this study analyzed previously published literature, ethical approval was not required.

### 2.3 Inclusion criteria

#### 2.3.1 Type of study

Only RCTs published in English or Chinese were eligible for inclusion.

#### 2.3.2 Participants

Participants were patients diagnosed with SAP according to the Nomenclature and Criteria for Diagnosis of Ischemic Heart Disease (ISFC/WHO, 1979) or the Guidelines for the Diagnosis and Treatment of Chronic Stable Angina (Interventional Cardiology Group, Chinese Society of Cardiology, Chinese Medical Associationn et al., 2018).

#### 2.3.3 Intervention and comparison

Conventional therapy (CT) consisted of antiplatelet agents, lipid-lowering agents, beta-blockers, RAAS inhibitors, and CCBs. Eligible studies were categorized into two comparisons: (1) CDP plus CT versus CT alone; and (2) CDP plus CT versus nitrates plus CT, with the same background CT administered in both groups. No restrictions were placed on the duration of CDP treatment.

#### 2.3.4 Outcomes

1. Primary outcomes: angina efficacy, electrocardiographic (ECG) efficacy, angina duration, angina frequency and nitroglycerine consumption.
2. Secondary outcomes: total cholesterol (TC), triglyceride (TG), low-density lipoprotein cholesterol (LDL-C), left ventricular ejection fraction (LVEF) and fibrinogen (FIB).

Angina efficacy was classified as follows: ① Markedly Effective: a reduction in the frequency of angina attacks by >80%; ② Effective: a reduction in the frequency of angina attacks by 50%–80%; ③ Ineffective: no reduction or worsening in the frequency of angina attacks. The overall response rate was calculated as follows: total angina efficacy = number of (markedly effective + effective) cases/total number of cases × 100% (Xing, 2025).

ECG efficacy was classified as follows: ① Markedly Effective: the resting ECG returns to normal or a nearly normal status; ② Effective: the resting ECG shows an elevation of the depressed ST-segment by >0.05 mV compared to baseline (but falls short of the normal level), or the inverted T-wave in primary leads becomes shallower by 25%; ③ Ineffective: no discernible changes are observed in the resting ECG compared to pre-treatment status. The overall response rate was calculated as follows: total ECG efficacy = number of (markedly effective + effective) cases/total number of cases × 100% (Cao, 2011).

3. Safety outcomes: the incidence and types of adverse events, serious adverse events, treatment discontinuations due to adverse events, and reported drug interactions.

Studies were included if they reported at least one prespecified outcome.

### 2.4 Exclusion criteria

1. Patients with comorbid conditions, such as malignant tumor, liver failure and renal failure were excluded.
2. For duplicate publications, only one was included.
3. Incomplete or inaccurate data.
4. Studies involving other TCM preparations or therapies in addition to CDP were excluded.

### 2.5 Information sources and search strategy

A comprehensive literature search was conducted in eight electronic databases [China National Knowledge Infrastructure (CNKI), Chinese Science and Technology Periodical Database (VIP), Wanfang Data, Chinese biomedical literature service system (SinoMed), PubMed, Excerpta Medica Database (Embase), Cochrane Library, Web of Science] to identify RCTs from inception to 31 May 2026. Additional manual searches of the reference lists of included studies were conducted to identify potentially eligible publications. The detailed search strategy for PubMed is provided as follows, and strategies for other databases can be found in Supplementary Appendix SI:

#1 “Angina, Stable” [MeSH Terms] OR “Anginas, Stable” [Title/Abstract] OR “Stable Angina” [Title/Abstract] OR “Stable Anginas” [Title/Abstract] OR “Angina Pectoris, Stable” [Title/Abstract] OR “Angina Pectori, Stable” [Title/Abstract] OR “Pectoris, Stable Angina” [Title/Abstract] OR “Pectori, Stable Angina” [Title/Abstract] OR “Stable Angina Pectori” [Title/Abstract] OR “Stable Angina Pectoris” [Title/Abstract] OR “Chronic Stable Angina” [Title/Abstract] OR “Angina, Chronic Stable” [Title/Abstract] OR “Anginas, Chronic Stable” [Title/Abstract] OR “Chronic Stable Anginas” [Title/Abstract] OR “Stable Angina, Chronic” [Title/Abstract] OR “Stable Anginas, Chronic” [Title/Abstract] OR “Angina Pectoris” [MeSH Terms] OR “Angor Pectoris” [Title/Abstract] OR “Stenocardia” [Title/Abstract] OR “Stenocardias” [Title/Abstract] OR “Stable Coronary Artery Disease” [Title/Abstract] OR “Chronic Coronary Syndromes” [Title/Abstract]
#2 “Compound Danshen Dripping Pill” [Title/Abstract] OR “Compound Danshen Pill” [Title/Abstract] OR “Compound Danshen Preparation” [Title/Abstract] OR “Guanxin Danshen Dripping Pill” [Title/Abstract] OR “Fufang Danshen” [Title/Abstract] OR “Fufang Danshen Dripping Pill” [Title/Abstract] OR “Fufang Danshen Diwan” [Title/Abstract] OR “Fufang Danshen Pill” [Title/Abstract] OR “Fufang Danshen Preparation” [Title/Abstract]
#3 “randomized controlled trial” [Publication type] OR “randomized clinical trial” [Publication type] OR “randomized trial” [Publication type] OR “clinical trial” [Publication type] OR “randomized controlled trial” [Title/Abstract] OR “randomized clinical trial” [Title/Abstract] OR “randomized trial” [Title/Abstract] OR “clinical trial” [Title/Abstract]
#4 #1 AND #2 AND #3

### 2.6 Study screening and data extraction

#### 2.6.1 General study characteristics

Screening of studies was conducted according to the predefined eligibility criteria: 1) Titles and abstracts were independently reviewed, and studies meeting the predefined eligibility criteria were included. 2) For studies requiring further evaluation, full-text articles were retrieved and assessed in detail.

Data were extracted using a standardized data extraction form, including study characteristics (author, publication year, study duration, sample size, patient age and sex, diagnostic criteria, disease duration, interventions, follow-up duration, and reported outcomes). When essential data were unavailable, attempts were made to contact the corresponding authors via email. Studies for which sufficient information could not be obtained were excluded. To provide a comprehensive evaluation of the efficacy and safety of CDP in patients with SAP, clinically relevant outcomes with relatively limited reporting were also considered. Screening and data extraction were conducted independently by two reviewers (ZC and WZ). Any discrepancies were resolved via consensus discussion or consultation with a third researcher.

#### 2.6.2 Assessment of CDP preparation characteristics

The characterization and reporting of CDP preparations in the included studies were assessed using the ConPhyMP guidelines and reporting tool (Heinrich et al., 2022). The assessment covered botanical identification, extraction and processing, product characteristics, phytochemical characterization, and analytical methods. Pharmacopoeial and official product information was used only to describe the reference formulation. Trial-level assessments relied solely on the original RCT reports; missing information was recorded as “not reported,” and designation as CDP alone was not considered evidence of compliance with a specific standard or comparability across trials. Two reviewers (WL and YZ) independently conducted the assessment, and detailed results are provided in Supplementary Appendix SIII.

### 2.7 Quality assessment

The Cochrane Risk of Bias tool for randomized trials (RoB 2) was adopted by two investigators to assess the methodological quality of individual studies. Bias assessment was conducted according to the dedicated signaling questions across the following five domains: 1) randomization process, 2) deviations from the intended interventions, 3) missing outcome data, 4) measurement of the outcome, and 5) selection of the reported result. Each domain was rated as “low risk of bias,” “some concerns,” or “high risk of bias.” The overall risk of bias judgment for each study was determined according to the assessments across all domains (Sterne et al., 2019).

### 2.8 Statistical analysis

A meta-analysis was performed with the Review Manager software (Cochrane Collaboration, version 5.4) along with Stata 18.0. Dichotomous outcomes were expressed as risk ratios (RRs) with 95% confidence intervals (CIs). For continuous outcomes, mean differences (MDs) were used when outcomes were measured using the identical scales and units across studies, while standardized mean differences (SMDs) were applied for outcomes evaluating the same construct with different measurement scales or units. Statistical heterogeneity was assessed using the I^2^ statistic and Chi-square test, with I^2^ > 50% indicating substantial heterogeneity. A random-effects model was used when substantial heterogeneity was present; otherwise, a fixed-effect model was applied (Higgins et al., 2003). Statistical significance was defined as a two-tailed p < 0.05. Subgroup analyses were performed according to treatment duration to explore potential sources of heterogeneity, and differences between subgroups were assessed using the test for subgroup differences. Sensitivity analyses were conducted using the leave-one-out method by sequentially excluding individual studies from the pooled analyses to evaluate the robustness of the findings. Funnel plots were generated to evaluate potential publication bias when at least 10 studies were available for a specific outcome.

### 2.9 Certainty of evidence

The GRADE (Grading of Recommendations Assessment, Development, and Evaluation) tool was adopted to assess the certainty of evidence for each outcome (Meerpohl et al., 2012). Evidence profiles were generated with the GRADEpro tool (http://gdt.guidelinedevelopment.org). The assessment accounted for study design, methodological limitations, inconsistency, indirect evidence, imprecision, and additional factors. The certainty of evidence was categorized as high, moderate, low, or very low according to the GRADE criteria.

## 3 Results

### 3.1 Study screening

A total of 5,974 records were identified through database searches. After removal of 3,076 duplicates, 2,898 records underwent title and abstract screening, resulting in the exclusion of 1,655 irrelevant articles. The remaining 1,243 records were retrieved for full-text assessment. During the full-text review, 1,150 articles were excluded for the following reasons: 1) Not RCTs (n = 142); 2) Ineligible study population (n = 632); 3) Ineligible outcome (n = 9); 4) Duplicate publication (n = 65); 5) Full-text unavailable (n = 78); 6) NOT journal articles (n = 6); 7) Ineligible intervention (n = 218). A final total of 93 articles were retained for the systematic review (Shi, 1997; Li et al., 1998; Zhou, 1998; Feng et al., 1999; Li et al., 1999; Zhang et al., 1999; Zhu, 1999; Wang et al., 2000; Wu and Liu, 2000; Dong and Zhang, 2001; Guo et al., 2001; Li and Jin, 2001; Shi et al., 2001; Tan and Liu, 2001; Xue and Liu, 2001; Yang et al., 2001; Zhao, 2001; Zhu, 2001; Deng et al., 2002; Feng and Gao, 2002; Pan, 2002; Yao et al., 2002; Yao, 2002; Chu and Geng, 2003; Liu and Li, 2003; Li et al., 2003; Wang et al., 2003; Wu and Guo, 2003; Yuan, 2003; Huang et al., 2004; Jiao, 2005; Li and Yang, 2005; Yao et al., 2005; Zou, 2005; Li et al., 2006; Qiao et al., 2006; Chen and Zhu, 2007; Chi et al., 2007; Wang and Chen, 2007; Zheng, 2007; Zhuang, 2007; Feng, 2008; Ma S. Q., 2008; Ma Y. L., 2008; Pan, 2008; Wang, 2008; Zhang and Zhu, 2008; Wang, 2009; Wei and Han, 2009; Xu, 2009; Yang and Li, 2009; Zhang, 2009; Zhao, 2009; Lin and Fang, 2010; Pu and Shen, 2010; Cao, 2011; Feng, 2011; Jiang et al., 2011; Pang and Li, 2011; Song and Gao, 2011; Sun and Suo, 2011; Yang, 2011; Zhang, 2011; He et al., 2012; Liang et al., 2012; Li, 2012; Shao, 2012; Wu and Yu, 2012; Wu et al., 2012; Gong, 2013; Liu et al., 2013; Shao et al., 2013; Zhang, 2013; Wang and Chen, 2014; Zhang, 2014; Zhao, 2015; Wang et al., 2017; Zhang, 2018; Dong, 2019; Li, 2019; Qu, 2019; Ma, 2020; Zhao, 2020; Zheng, 2020; Kong, 2021; Wang et al., 2021; Wang et al., 2022; Qin et al., 2024; Tang, 2024; Wang and Ju, 2024; Deng et al., 2025; Gu et al., 2025; Xing, 2025). Ultimately, 93 RCTs met the eligibility criteria and were included in the systematic review and meta-analysis. The study selection process is presented in Figure 1.

**FIGURE 1:**
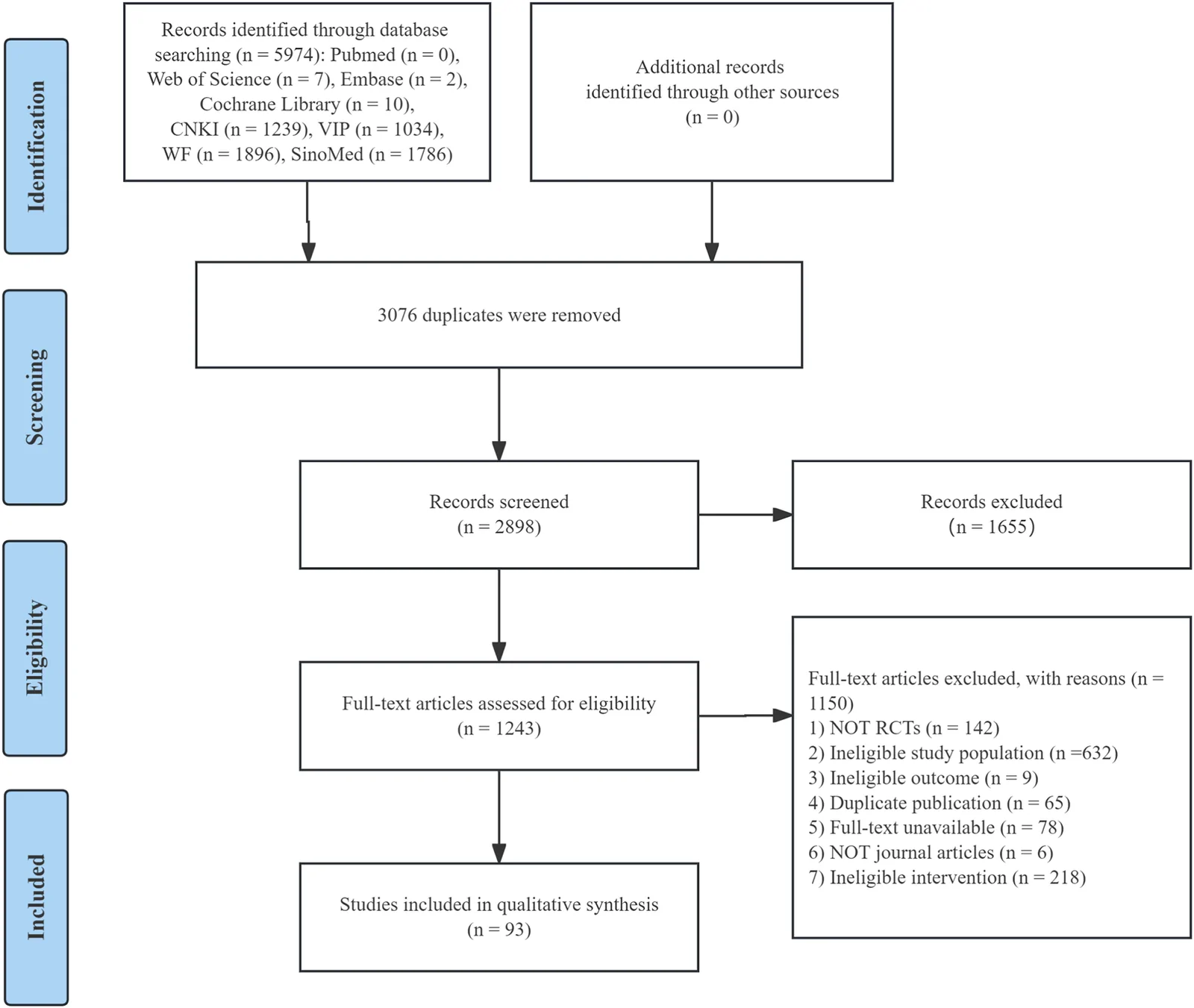
Flow diagram depicting the study screening process.

### 3.2 Study characteristics

The characteristics of all included studies are summarized in Supplementary Table S1. A total of 93 RCTs published between 1997 and 2025 were included, involving 10,208 participants. Among these, 26 RCTs (2,564 participants) compared CDP plus CT with CT alone, whereas 67 RCTs (7,644 participants) compared CDP plus CT with nitrates plus CT. The treatment duration ranged from 14 to 180 days. No significant differences in baseline characteristics were observed among the included studies.

### 3.3 Characterization of CDP preparations

The reporting and characterization of CDP preparations varied substantially across the included RCTs. All 93 studies met the ConPhyMP criterion for title and abstract reporting; however, none reported botanical or taxonomic authentication or provided sufficient information on extraction and processing procedures. Only one study (1.1%) fulfilled the criterion for reporting the characteristics of the finished commercial product. All studies involved botanical drugs covered by pharmacopoeial monographs, and 76 (81.7%) reported information on bioactive or analytical marker metabolites. Manufacturer and certificate-related information, as defined by the corresponding ConPhyMP item, was reported in 51 studies (54.8%). In contrast, detailed analytical characterization was largely absent: none of the included RCTs reported triple chemical fingerprinting, a single chemical fingerprinting approach meeting the ConPhyMP criteria, or comparison with official reference standards. Detailed item-level results are presented in Supplementary Appendix SIII.

### 3.4 Risk of bias assessment

The risk-of-bias assessment using RoB 2 is presented in Figure 2. Overall, 71 RCTs were judged as having some concerns and 22 as having a high risk of bias. The main methodological limitations were related to the randomization process and deviations from the intended interventions. Although all included studies reported random allocation, details regarding sequence generation and allocation concealment were frequently insufficient. Blinding of participants, personnel, and outcome assessors was also inadequately reported in many trials, raising concerns particularly for outcomes susceptible to subjective assessment. Missing outcome data were generally limited based on the information reported in the included studies. In addition, none of the 93 RCTs had an identified prespecified protocol or trial registration; therefore, the possibility of selective reporting could not be excluded.

**FIGURE 2:**
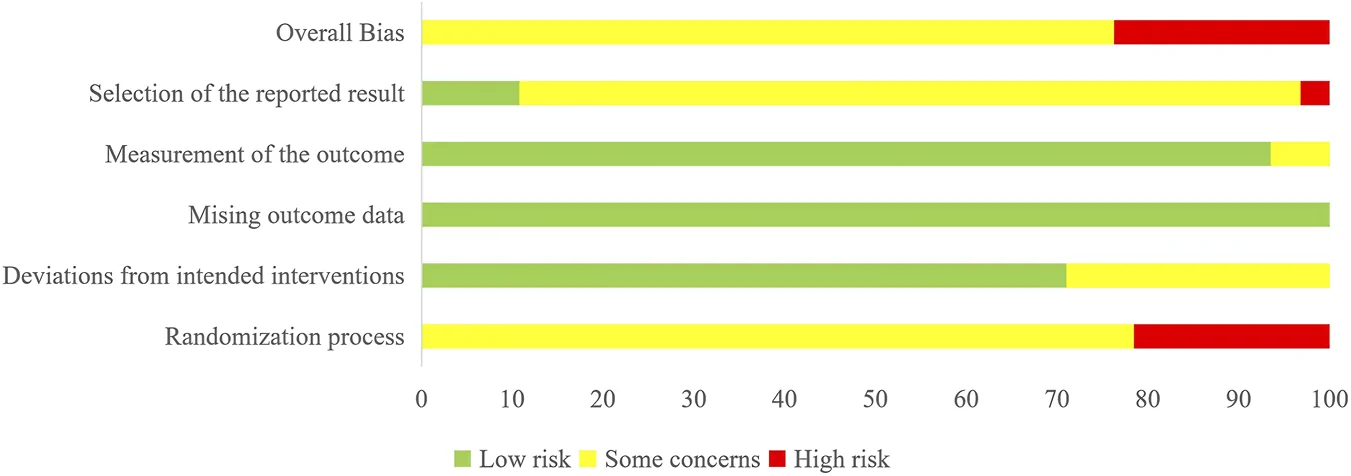
Risks of bias of the included studies.

### 3.5 Meta-analysis results

#### 3.5.1 CDP + CT vs. CT

##### 3.5.1.1 Angina efficacy

Twelve RCTs involving 1,020 participants evaluated angina efficacy (Pan, 2002; Wang, 2008; Feng, 2011; Zhang, 2011; Li, 2012; Wu and Yu, 2012; Liu et al., 2013; Zhang, 2014; Li, 2019; Ma, 2020; Gu et al., 2025; Xing, 2025) (Figure 3A). No significant heterogeneity was observed among the included studies (*I* ^*2*^ = 0%, *p* = 0.63), therefore, a fixed-effect model was applied. The pooled RR was 1.26 (95% CI: 1.19–1.33).

**FIGURE 3:**
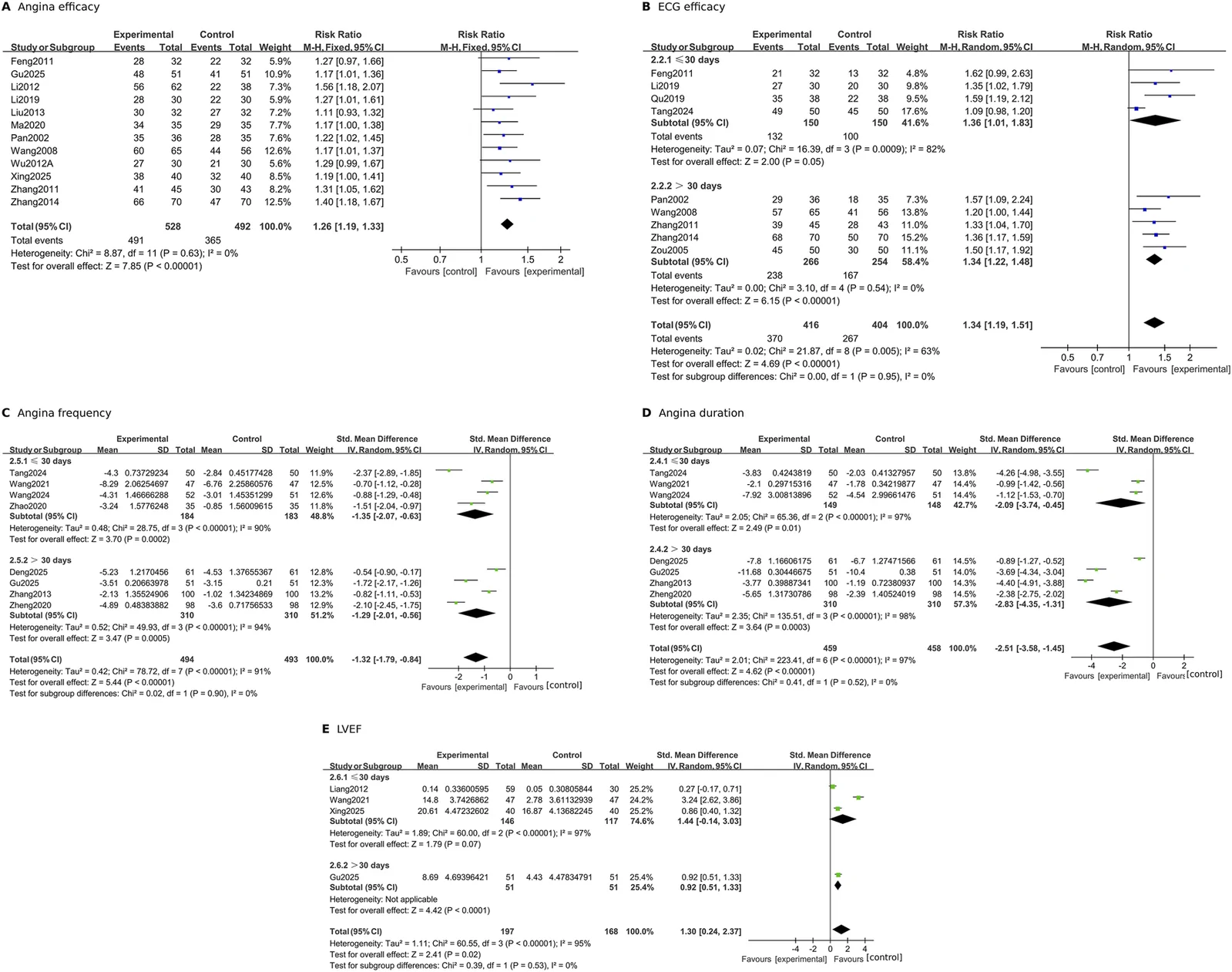
Meta-analysis results of compound danshen dripping Pills plus conventional therapy versus conventional therapy alone: **(A)** angina efficacy; **(B)** ECG efficacy; **(C)** angina frequency; **(D)** angina duration; and **(E)** left ventricular ejection fraction.

##### 3.5.1.2 ECG efficacy

Nine RCTs involving 820 participants evaluated ECG efficacy (Pan, 2002; Zou, 2005; Wang, 2008; Feng, 2011; Zhang, 2011; Zhang, 2014; Li, 2019; Qu, 2019; Tang, 2024) (Figure 3B). Substantial heterogeneity was observed (*I* ^*2*^ = 63%), which persisted in the sensitivity analysis; therefore, a random-effects model was used. The overall pooled RR was 1.34 (95% CI: 1.19–1.51). In subgroup analyses by treatment duration, the pooled RR was 1.36 (95% CI: 1.01 to 1.83, *p* = 0.05) in the ≤30-day subgroup (4 RCTs, n = 300) and 1.34 (95% CI: 1.22 to 1.48, *p* < 0.00001) in the >30-day subgroup (5 RCTs, n = 520). The test for subgroup differences was not statistically significant (*p* = 0.95).

##### 3.5.1.3 Angina frequency

Eight RCTs involving 987 participants evaluated angina frequency (Zhang, 2013; Zhao, 2020; Zheng, 2020; Wang et al., 2021; Tang, 2024; Wang and Ju, 2024; Deng et al., 2025; Gu et al., 2025) (Figure 3C). Considerable heterogeneity was observed (*I* ^*2*^ = 91%); therefore, a random-effects model was used. The overall pooled SMD was −1.32 (95% CI: −1.79 to −0.84). In subgroup analyses by treatment duration, the pooled SMD was −1.35 (95% CI: −2.07 to −0.63, *p* = 0.0002) in the ≤30-day subgroup and −1.29 (95% CI: −2.01 to −0.56, *p* = 0.0005) in the >30-day subgroup. The test for subgroup differences was not statistically significant (*p* = 0.90).

##### 3.5.1.4 Angina duration

Seven RCTs involving 917 participants evaluated angina duration (Zhang, 2013; Zheng, 2020; Wang et al., 2021; Tang, 2024; Wang and Ju, 2024; Deng et al., 2025; Gu et al., 2025) (Figure 3D). Considerable heterogeneity was observed (*I* ^*2*^ = 97%); therefore, a random-effects model was used. The overall pooled SMD was −2.51 (95% CI: −3.58 to −1.45). In subgroup analyses by treatment duration, the pooled SMD was −2.09 (95% CI: −3.74 to −0.45, *p* = 0.01) in the ≤30-day subgroup and −2.83 (95% CI: −4.35 to −1.31, *p* = 0.0003) in the >30-day subgroup. The test for subgroup differences was not statistically significant (*p* = 0.52).

##### 3.5.1.5 Left ventricular ejection fraction

Four RCTs involving 365 participants evaluated LVEF (Liang et al., 2012; Wang et al., 2021; Gu et al., 2025; Xing, 2025) (Figure 3E). Considerable heterogeneity was observed (*I* ^*2*^ = 95%); therefore, a random-effects model was used. The overall pooled SMD was 1.30 (95% CI: 0.24–2.37). In subgroup analyses by treatment duration, the pooled SMD was 1.44 (95% CI: −0.14 to 3.03, *p* = 0.07) in the ≤30-day subgroup and 0.92 (95% CI: 0.51–1.33, *p* < 0.0001) in the >30-day subgroup. The test for subgroup differences was not statistically significant (*p* = 0.53).

##### 3.5.1.6 Other outcomes

Additional outcomes were also evaluated to comprehensively assess the therapeutic effects of CDP, including TCM syndrome score [chest pain score (Tang, 2024; Deng et al., 2025; Xing, 2025), chest tightness score (Deng et al., 2025; Xing, 2025) and palpitation score (Tang, 2024)], blood lipids [total cholesterol (TC), triglyceride (TG) and low-density lipoprotein cholesterol (LDL-C) (Liang et al., 2012)], angina efficacy [nitroglycerine consumption (Zhao, 2020) and 6-min walk test (6MWT) (Xing, 2025)], coagulation function [fibrinogen (FIB) (Liang et al., 2012; Dong, 2019)], ST-segment depression temporal burden indices [number of ST-segment depressions (Qin et al., 2024), duration of ST-segment depression (Qin et al., 2024), maximum ST segment depression (Stmax) (Zhang, 2013) and number of leads with ST segment depression (NST) (Zhao, 2020)], inflammatory factors [C-reactive protein (CRP) (Gu et al., 2025)], endothelial function [endothelin-1 (ET-1), nitric oxide (NO) (Qin et al., 2024)] and antioxidant capacity [superoxide dismutase (SOD) (Qin et al., 2024)]. The corresponding effect estimates, 95% CIs, heterogeneity statistics, and numbers of included studies are presented in Table 1.

**TABLE 1:** Meta-analysis results of other outcomes (Compound Danshen Dripping Pills + conventional therapy vs. conventional therapy).

Classification	Outcome	Number of studies	Sample size (T/C)	Measures	Effect estimate (95% CI)	Heterogeneity (*I* ^*2*^)	*p*
TCM syndrome score	Chest pain score	3	151/151	MD (Random)	−0.54 [-0.80, −0.28]	95%	<0.00001
​	Chest tightness score	2	101/101	MD (Random)	−0.55 [-0.88, −0.22]	83%	0.02
​	Palpitation score	1	50/50	MD (N/A)	−0.44 [-0.48, −0.40]	—	—
Blood Lipids	TC	1	59/30	MD (N/A)	−2.13 [-2.56, −1.70]	—	—
​	TG	1	59/30	MD (N/A)	−1.45 [-1.94, −0.96]	—	—
​	LDL-C	1	59/30	MD (N/A)	−1.33 [-1.80, −0.86]	—	—
Angina efficacy	Nitroglycerine consumption	1	35/35	MD (N/A)	−0.90 [-1.12, −0.68]	—	—
​	6MWT	1	40/40	MD (N/A)	28.72 [5.95, 51.49]	—	—
Coagulation function	FIB	2	107/78	SMD (Random)	−2.58 [-3.53, −1.62]	82%	0.02
ST-segment depression temporal burden indices	Number of ST-segment depressions	1	49/49	MD (N/A)	−1.28 [-1.83, −0.73]	—	—
​	Duration of ST-segment depression	1	49/49	MD (N/A)	−1.36 [-1.82, −0.90]	—	—
​	Stmax	1	100/100	MD (N/A)	−0.38 [-0.52, −0.24]	—	—
​	NST	1	35/35	MD (N/A)	−0.64 [-0.90, −0.38]	—	—
Inflammatory factors	CRP	1	51/51	MD (N/A)	−3.62 [-4.69, −2.55]	—	—
Endothelial function	ET-1	1	49/49	MD (N/A)	−14.00 [-15.21, −12.79]	—	—
​	NO	1	49/49	MD (N/A)	13.21 [11.19, 15.23]	—	—
Antioxidant capacity	SOD	1	49/49	MD (N/A)	8.17 [6.39, 9.95]	—	—

Abbreviations: CI, confidence interval; *I* ^*2*^, heterogeneity; TC, total cholesterol; TG, triglyceride; LDL-C, low-density lipoprotein cholesterol; 6MWT, 6-min walk test; FIB, fibrinogen; Stmax, maximum ST, segment depression; NST, number of leads with ST, segment depression; CRP, C-reactive protein; ET-1, endothelin-1; NO, nitric oxide; SOD, superoxide dismutase.

##### 3.5.1.7 Adverse events

Ten studies (Lin and Fang, 2010; Zhang, 2011; Dong, 2019; Zheng, 2020; Wang et al., 2022; Tang, 2024; Wang and Ju, 2024; Deng et al., 2025; Gu et al., 2025; Xing, 2025) reported adverse events. Adverse events occurred in 55 of 551 participants (10.0%) in the CDP plus CT group and 65 of 548 participants (11.9%) in the CT group. Reported events included headache, dizziness or vertigo, fatigue, drowsiness, gastrointestinal symptoms, hypotension, increased heart rate, palpitations, flushing, and abnormalities in liver or renal function and routine blood tests (Supplementary Appendix SIV). The pooled analysis showed no statistically significant between-group difference in the incidence of adverse events (RR = 0.84, 95% CI: 0.60 to 1.18; *p* = 0.31; *I* ^*2*^ = 0%).

##### 3.5.1.8 Sensitivity analyses

Leave-one-out sensitivity analyses showed that sequential exclusion of individual studies did not materially alter the pooled effect estimates. Detailed sensitivity analysis data are available in Supplementary Appendix SII.

##### 3.5.1.9 Risk of publication bias

Funnel plots were generated for angina efficacy (Figure 4A) and adverse events (Figure 4B). The funnel plot for angina efficacy showed slight asymmetry, whereas that for adverse events appeared relatively symmetrical.

**FIGURE 4:**
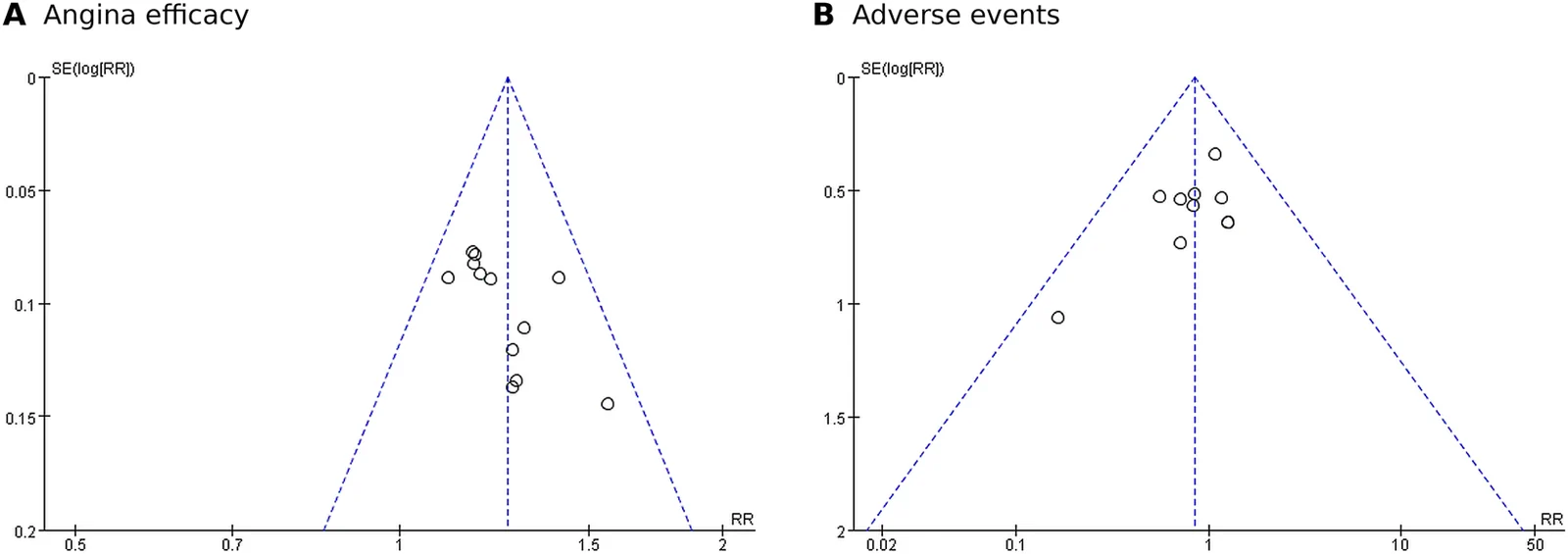
Funnel plots for Compound Danshen Dripping Pills plus conventional therapy versus conventional therapy alone: **(A)** angina efficacy and **(B)** adverse events.

##### 3.5.1.10 Certainty of evidence

According to the GRADE framework, the certainty of evidence varied across the evaluated outcomes (Table 2). Evidence for angina efficacy was rated as moderate because of serious risk of bias. Evidence for ECG efficacy was rated as low because of serious risk of bias and serious inconsistency. Evidence for adverse events was rated as low because of serious risk of bias and serious imprecision. Evidence for angina frequency and angina duration was rated as very low because of serious risk of bias and very serious inconsistency. No downgrading was applied for indirectness or publication bias.

**TABLE 2:** Summary of the study findings (Compound Danshen Dripping Pills + conventional therapy vs. conventional therapy).

Outcome	Quality assessment							No. of patients		Effect		Certainty	Importance
	No. of studies	Design	Risk of bias	Inconsistency	Indirectness	Imprecision	Publication bias	T	C	Relative (95% CI)	Absolute (95% CI)		
Angina efficacy	12	RCT	Serious	Not serious	Not serious	Not serious	N/A	491/528 (93.0%)	365/492 (74.2%)	RR 1.26 (1.19–1.33)	N/A	⊕⊕⊕O Moderate	Critical
ECG efficacy	9	RCT	Serious	Serious	Not serious	Not serious	N/A	370/416 (88.9%)	267/404 (66.1%)	RR 1.34 (1.19–1.51)	N/A	⊕⊕OO Low	Critical
Adverse Events	10	RCT	Serious	Not serious	Not serious	Serious	N/A	55/551 (10%)	65/548 (11.9%)	RR 0.84 (0.6–1.18)	N/A	⊕⊕OO Low	Critical
Angina duration	7	RCT	Serious	Very serious	Not serious	Not serious	N/A	459	458	N/A	SMD 2.51 lower (3.58–1.45 lower)	⊕OOO Very low	Critical
Angina frequency	8	RCT	Serious	Very serious	Not serious	Not serious	N/A	494	493	N/A	SMD 1.32 lower (1.79–0.84 lower)	⊕OOO Very low	Critical

Abbreviations: CI, confidence interval; SMD, std mean difference.

#### 3.5.2 CDP + CT vs. Nitrates + CT

##### 3.5.2.1 Angina efficacy

Fifty-six RCTs involving 6,589 participants reported angina efficacy (Li et al., 1998; Zhou, 1998; Feng et al., 1999; Zhang et al., 1999; Zhu, 1999; Wang et al., 2000; Wu and Liu, 2000; Dong and Zhang, 2001; Guo et al., 2001; Li and Jin, 2001; Shi et al., 2001; Tan and Liu, 2001; Xue and Liu, 2001; Yang et al., 2001; Zhao, 2001; Zhu, 2001; Deng et al., 2002; Feng and Gao, 2002; Yao et al., 2002; Yao, 2002; Chu and Geng, 2003; Liu and Li, 2003; Li et al., 2003; Wang et al., 2003; Yuan, 2003; Jiao, 2005; Li and Yang, 2005; Yao et al., 2005; Li et al., 2006; Qiao et al., 2006; Chen and Zhu, 2007; Chi et al., 2007; Wang and Chen, 2007; Zheng, 2007; Zhuang, 2007; Feng, 2008; Ma S. Q., 2008; Ma Y. L., 2008; Pan, 2008; Zhang and Zhu, 2008; Wang, 2009; Wei and Han, 2009; Xu, 2009; Yang and Li, 2009; Zhang, 2009; Cao, 2011; Jiang et al., 2011; Pang and Li, 2011; Song and Gao, 2011; Sun and Suo, 2011; He et al., 2012; Gong, 2013; Shao et al., 2013; Wang and Chen, 2014; Zhao, 2015) (Figure 5A). Substantial heterogeneity was observed (I^2^ = 65%), and a random-effects model was used. The overall pooled estimate was RR = 1.11 (95% CI: 1.08–1.15). In subgroup analyses by treatment duration, the pooled RR was 1.09 (95% CI 1.04 to 1.13; *p* < 0.0001) for treatment duration ≤30 days (24 RCTs, n = 2,518) and 1.13 (95% CI 1.08 to 1.19; *p* < 0.00001) for duration >30 days (32 RCTs, n = 4,071). The test for subgroup differences was not statistically significant (*p* = 0.19).

**FIGURE 5:**
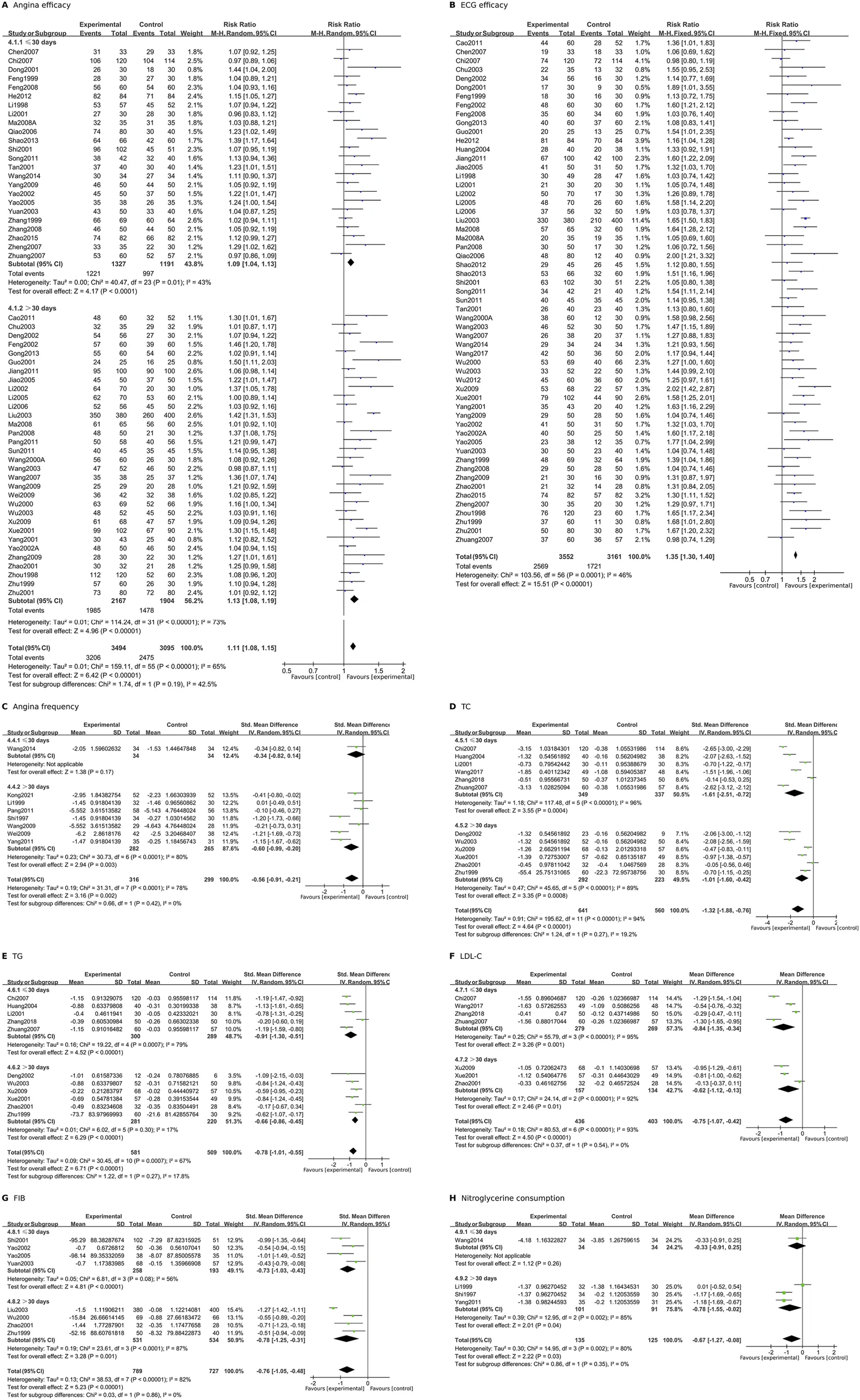
Meta-analysis results of compound danshen dripping pills plus conventional therapy versus nitrates plus conventional therapy: **(A)** angina efficacy; **(B)** ECG efficacy; **(C)** angina frequency; **(D)** total cholesterol; **(E)** triglycerides; **(F)** low-density lipoprotein cholesterol; **(G)** fibrinogen; and **(H)** nitroglycerin consumption.

##### 3.5.2.2 ECG efficacy

Fifty-seven RCTs involving 6,713 participants reported ECG efficacy (Li et al., 1998; Zhou, 1998; Feng et al., 1999; Zhang et al., 1999; Zhu, 1999; Wang et al., 2000; Wu and Liu, 2000; Dong and Zhang, 2001; Guo et al., 2001; Li and Jin, 2001; Shi et al., 2001; Tan and Liu, 2001; Xue and Liu, 2001; Yang et al., 2001; Zhao, 2001; Zhu, 2001; Deng et al., 2002; Feng and Gao, 2002; Yao et al., 2002; Yao, 2002; Chu and Geng, 2003; Liu and Li, 2003; Li et al., 2003; Wang et al., 2003; Wu and Guo, 2003; Yuan, 2003; Huang et al., 2004; Jiao, 2005; Li and Yang, 2005; Yao et al., 2005; Li et al., 2006; Qiao et al., 2006; Chen and Zhu, 2007; Chi et al., 2007; Wang and Chen, 2007; Zheng, 2007; Zhuang, 2007; Feng, 2008; Ma S. Q., 2008; Ma Y. L., 2008; Pan, 2008; Zhang and Zhu, 2008; Xu, 2009; Yang and Li, 2009; Zhang, 2009; Cao, 2011; Jiang et al., 2011; Song and Gao, 2011; Sun and Suo, 2011; He et al., 2012; Shao, 2012; Wu et al., 2012; Gong, 2013; Shao et al., 2013; Wang and Chen, 2014; Zhao, 2015; Wang et al., 2017) (Figure 5B). Moderate heterogeneity was observed (*I* ^*2*^ = 46%), and a fixed-effect model was used. The pooled RR was 1.35 (95% CI: 1.30 to 1.40, *p* < 0.00001).

##### 3.5.2.3 Angina frequency

Eight RCTs involving 615 participants reported angina frequency (Shi, 1997; Li et al., 1999; Wang, 2009; Wei and Han, 2009; Pang and Li, 2011; Yang, 2011; Wang and Chen, 2014; Kong, 2021) (n = 615; Figure 5C). Substantial heterogeneity was observed (*I* ^*2*^ = 78%), and a random-effects model was used. The pooled SMD was −0.56 (95% CI: −0.91 to −0.21). In subgroup analyses by treatment duration, the SMD was −0.34 (95% CI: −0.82 to 0.14) for duration ≤30 days (1 RCT, n = 68) and −0.60 (95% CI: −0.99 to −0.20, *p* = 0.003) for duration >30 days (7 RCTs, n = 547). The test for subgroup differences was not statistically significant (*p* = 0.42).

##### 3.5.2.4 Total cholesterol

Twelve RCTs involving 1,201 participants reported TC (Zhu, 1999; Li and Jin, 2001; Xue and Liu, 2001; Zhao, 2001; Deng et al., 2002; Wu and Guo, 2003; Huang et al., 2004; Chi et al., 2007; Zhuang, 2007; Xu, 2009; Wang et al., 2017; Zhang, 2018) (Figure 5D). Substantial heterogeneity was observed (*I* ^*2*^ = 94%), and a random-effects model was used. The pooled SMD was −1.32 (95% CI: −1.88 to −0.76). In subgroup analyses by treatment duration, the SMD was −1.61 (95% CI: −2.51 to −0.72, *p* = 0.0004) for the ≤30-day subgroup (6 RCTs, n = 686) and −1.01 (95% CI: −1.60 to −0.42, *p* = 0.0008) for the >30-day subgroup (6 RCTs, n = 515). The test for subgroup differences was not statistically significant (*p* = 0.27).

##### 3.5.2.5 Triglyceride

Eleven RCTs involving 1,090 participants provided data on TG (Zhu, 1999; Li and Jin, 2001; Xue and Liu, 2001; Zhao, 2001; Deng et al., 2002; Wu and Guo, 2003; Huang et al., 2004; Chi et al., 2007; Zhuang, 2007; Xu, 2009; Zhang, 2018) (Figure 5E). Substantial heterogeneity was observed (*I* ^*2*^ = 67%), and a random-effects model was used. The pooled SMD was −0.78 (95% CI: −1.01 to −0.55). In subgroup analyses by treatment duration, the SMD was −0.91 (95% CI: −1.30 to −0.51, *p* < 0.00001) for the ≤30-day subgroup (5 RCTs, n = 589) and −0.66 (95% CI: −0.86 to −0.45, *p* < 0.00001) for the >30-day subgroup (6 RCTs, n = 501). The test for subgroup differences was not statistically significant (*p* = 0.27).

##### 3.5.2.6 Low-density lipoprotein cholesterol

Seven RCTs involving 839 participants provided data on LDL-C (Xue and Liu, 2001; Zhao, 2001; Chi et al., 2007; Zhuang, 2007; Xu, 2009; Wang et al., 2017; Zhang, 2018) (n = 839; Figure 5F). Substantial heterogeneity was observed (*I* ^*2*^ = 93%), and a random-effects model was used. The pooled MD was −0.75 (95% CI: −1.07 to −0.42). In subgroup analyses by treatment duration, the MD was −0.84 (95% CI: −1.35 to −0.34, *p* = 0.001) for the ≤30-day subgroup (4 RCTs, n = 548) and −0.62 (95% CI −1.12 to −0.13, *p* = 0.01) for the >30-day subgroup (3 RCTs, n = 291). The test for subgroup differences was not statistically significant (*p* = 0.54).

##### 3.5.2.7 Fibrinogen

Eight RCTs involving 1,516 participants provided data on FIB (Zhu, 1999; Wu and Liu, 2000; Shi et al., 2001; Zhao, 2001; Yao, 2002; Liu and Li, 2003; Yuan, 2003; Yao et al., 2005) (n = 1,516; Figure 5G). Substantial heterogeneity was observed (*I* ^*2*^ = 82%), and a random-effects model was used. The pooled SMD was −0.76 (95% CI: −1.05 to −0.48). In subgroup analyses by treatment duration, the SMD was −0.73 (95% CI: −1.03 to −0.43, *p* < 0.00001) for the ≤30-day subgroup (4 RCTs, n = 451) and −0.78 (95% CI: −1.25 to −0.31, *p* = 0.001) for the >30-day subgroup (4 RCTs, n = 1,065). The test for subgroup differences was not statistically significant (*p* = 0.86).

##### 3.5.2.8 Nitroglycerine consumption

Four RCTs involving 260 participants provided data on nitroglycerin consumption (Shi, 1997; Li et al., 1999; Yang, 2011; Wang and Chen, 2014) (n = 260; Figure 5H). Substantial heterogeneity was observed (*I* ^*2*^ = 80%), and a random-effects model was used. The pooled SMD was −0.67 (95% CI: −1.27 to −0.08). In subgroup analyses by treatment duration, the SMD was −0.33 (95% CI: −0.91 to 0.25, *p* = 0.26) for the ≤30-day subgroup (1 RCT, n = 68) and −0.78 (95% CI: −1.55 to −0.02, *p* = 0.04) for the >30-day subgroup (3 RCTs, n = 192). The test for subgroup differences was not statistically significant (*p* = 0.35).

##### 3.5.2.9 Other outcomes

Additional outcomes were also evaluated to comprehensively assess the therapeutic effects of CDP, including angina efficacy [angina duration (Wang, 2009; Wei and Han, 2009; Kong, 2021)], exercise stress test parameters [RPP (Shi, 1997; Wang and Chen, 2007; Yang, 2011) and time to exercise-induced angina (Shi, 1997; Li et al., 1998; Yang, 2011)], ST-segment depression temporal burden indices [number of ST-segment depressions (Shi, 1997; Li et al., 1999; Yang, 2011), duration of ST-segment depression (Shi, 1997; Li et al., 1999; Li et al., 2003; Yang, 2011), Stmax (Li et al., 1999; Li et al., 2003), ΣST (Wang and Chen, 2007), and time to ≥1 mm ST segment depression (Shi, 1997; Yang, 2011)], inflammatory factors [CRP (Wang et al., 2017)], ventricular systolic function [LVEF (Shi, 1997; Wang and Chen, 2007; Yang, 2011)], and endothelial function [ ET-1 and NO (Li and Jin, 2001; Pu and Shen, 2010)]. Detailed results are presented in Table 3.

**TABLE 3:** Meta-analysis results of other outcomes (Compound Danshen Dripping Pills + conventional therapy vs. Nitrates + conventional therapy).

Classification	Outcome	Number of studies	Sample size (T/C)	Measures	Effect estimate (95% CI)	Heterogeneity (*I* ^*2*^)	*p*
Angina efficacy	Angina duration	3	123/118	SMD(Fixed)	−0.68 [−0.94, −0.42]	3%	0.36
Exercise stress test parameters	RPP	3	107/98	MD (Random)	−4572.65 [−5423.98, −3721.33]	97%	<0.00001
​	Time to exercise-induced angina	3	118/108	MD (Fixed)	19.92 [−4.95, 44.79]	47%	0.15
ST-segment depression temporal burden indices	Number of ST-segment depressions	3	101/91	MD (Random)	−44.58 [−94.87, 5.71]	100%	<0.00001
​	Duration of ST-segment depression	4	171/121	MD (Random)	−71.60 [−118.29, −24.92]	97%	<0.00001
​	Stmax	2	119/77	MD (Fixed)	−0.15 [−0.40, 0.10]	0%	0.81
​	ΣST	1	38/37	MD (N/A)	−0.60 [−0.82, −0.38]	—	—
​	Time to ≥1 mm ST segment depression	2	69/61	MD (Fixed)	91.54 [47.99, 135.08]	0%	0.84
Inflammatory factors	CRP	1	49/48	MD (N/A)	−0.74 [−1.12, −0.36]	—	—
Ventricular systolic function	LVEF	3	107/98	MD (Fixed)	6.49 [3.11, 9.87]	0%	0.61
Endothelial function	ET-1	2	80/80	SMD (Random)	−0.64 [−1.71, 0.43]	91%	0.001
​	NO	2	80/80	MD (Random)	2.84 [−1.13, 6.81]	73%	0.05

Abbreviations: CI, confidence interval; *I* ^*2*^, heterogeneity; RPP, peak rate–pressure product; Stmax, maximum ST, segment depression; ΣST, sum of ST, segment depression; CRP, C-reactive protein; LVEF, left ventricular ejection fraction; ET-1, endothelin-1; NO, nitric Oxide.

##### 3.5.2.10 Adverse events

46 studies (Shi, 1997; Li et al., 1998; Zhou, 1998; Li et al., 1999; Zhang et al., 1999; Zhu, 1999; Wang et al., 2000; Dong and Zhang, 2001; Li and Jin, 2001; Shi et al., 2001; Xue and Liu, 2001; Yang et al., 2001; Zhao, 2001; Feng and Gao, 2002; Yao, 2002; Wang et al., 2003; Wu and Guo, 2003; Huang et al., 2004; Jiao, 2005; Li and Yang, 2005; Li et al., 2006; Qiao et al., 2006; Chen and Zhu, 2007; Chi et al., 2007; Zheng, 2007; Zhuang, 2007; Ma S. Q., 2008; Pan, 2008; Wang, 2009; Wei and Han, 2009; Xu, 2009; Zhang, 2009; Zhao, 2009; Cao, 2011; Jiang et al., 2011; Pang and Li, 2011; Song and Gao, 2011; Sun and Suo, 2011; Yang, 2011; He et al., 2012; Shao, 2012; Wu and Yu, 2012; Gong, 2013; Shao et al., 2013; Zhao, 2015; Wang et al., 2017) reported adverse events. The reported adverse event rates were 2.8% (74/2,649) in the experimental group and 19.8% (457/2,312) in the control group. Reported adverse events in the experimental group mainly included gastrointestinal discomfort, whereas those in the control group mainly included headache, dizziness, gastrointestinal reactions, and facial flushing or burning sensation. Other reported events included palpitations, postural hypotension, and abnormal liver function (Supplementary Appendix SIV). Most reported adverse events were mild, although treatment discontinuation due to adverse events was reported in two studies. The pooled RR was 0.16 (95% CI: 0.13 to 0.20, *I* ^*2*^ = 36%, *p* < 0.00001).

##### 3.5.2.11 Sensitivity analyses

Leave-one-out sensitivity analyses did not materially alter the pooled effect estimates. Detailed results are provided in Supplementary Appendix SII.

##### 3.5.2.12 Risk of publication bias

Funnel plots were generated for angina efficacy (Figure 6A), ECG efficacy (Figure 6B), adverse events (Figure 6C), TC (Figure 6D), and TG (Figure 6E). Mild asymmetry was observed for angina efficacy and ECG efficacy, indicating possible small-study effects or publication bias. The funnel plots for adverse events, TC, and TG appeared relatively symmetrical, with no marked asymmetry observed.

**FIGURE 6:**
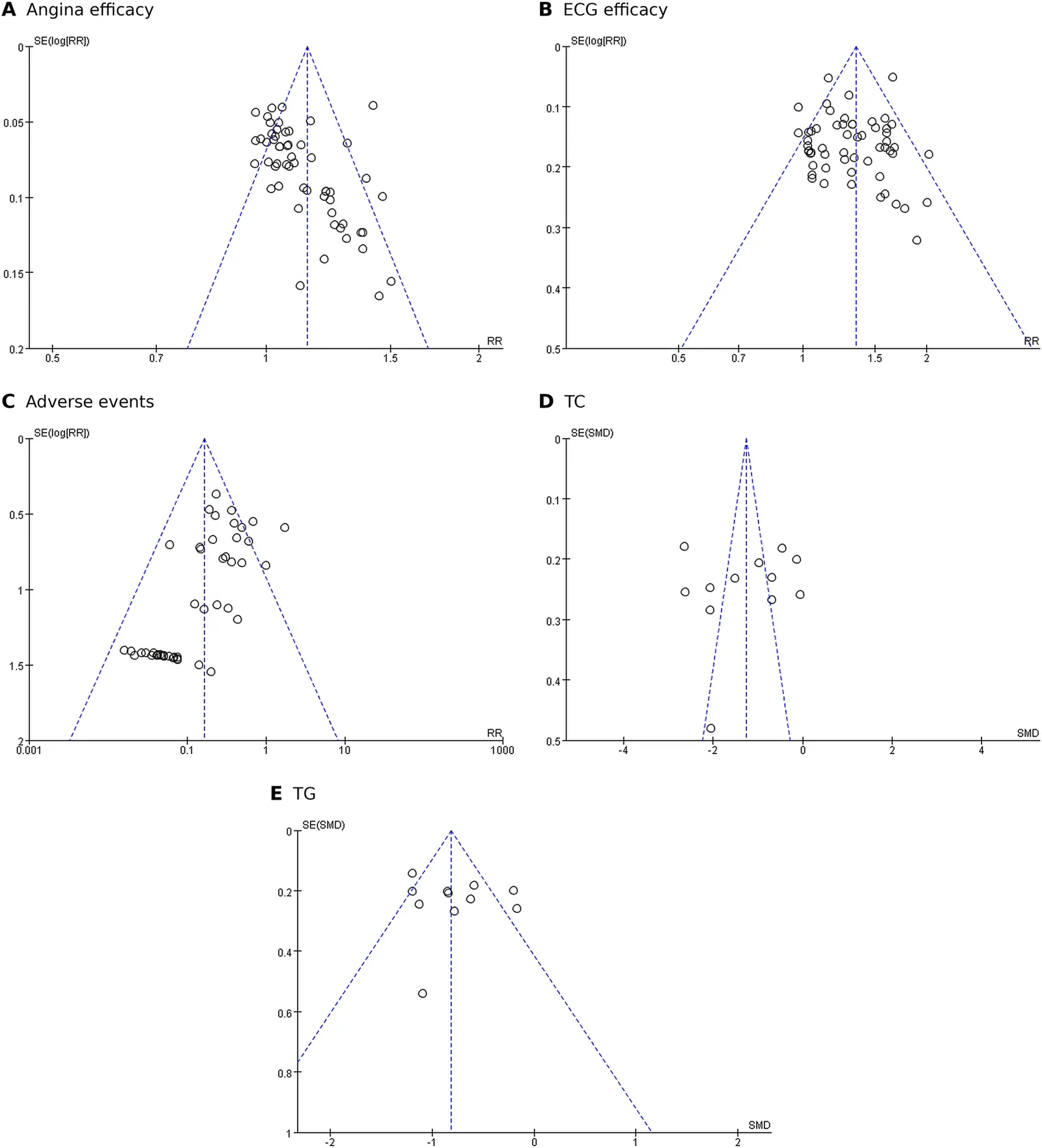
Funnel plots for compound danshen dripping pills plus conventional therapy versus nitrates plus conventional therapy: **(A)** angina efficacy; **(B)** ECG efficacy; **(C)** adverse events; **(D)** total cholesterol; and **(E)** triglycerides.

##### 3.5.2.13 Certainty of evidence

According to the GRADE framework, the certainty of evidence ranged from moderate to very low across the evaluated outcomes (Table 4). Evidence for ECG efficacy and adverse events was rated as moderate because of serious risk of bias. Evidence for angina efficacy and angina frequency was rated as low because of serious risk of bias and serious inconsistency. Evidence for TC, TG, LDL-C, and FIB was rated as very low because of serious risk of bias and very serious inconsistency. Evidence for nitroglycerin consumption was also rated as very low because of serious risk of bias, very serious inconsistency, and serious imprecision. No downgrading was applied for indirectness.

**TABLE 4:** Summary of the study findings (Compound Danshen Dripping Pills + conventional therapy vs. Nitrates + conventional therapy).

Outcome	Quality assessment							No. of patients		Effect		Certainty	Importance
	No. of studies	Design	Risk of bias	Inconsistency	Indirectness	Imprecision	Publication bias	T	C	Relative (95% CI)	Absolute (95% CI)		
Angina efficacy	56	RCT	Serious	Serious	Not serious	Not serious	N/A	3,206/3,494 (91.8%)	2,475/3,095 (80%)	RR 1.11 (1.08–1.15)	N/A	⊕⊕OO Low	Critical
ECG efficacy	57	RCT	Serious	Not serious	Not serious	Not serious	N/A	2,569/3,552 (72.3%)	1,721/3,161 (53.5%)	RR 1.35 (1.30–1.40)	N/A	⊕⊕⊕O Moderate	Critical
Adverse Events	46	RCT	Serious	Not serious	Not serious	Not serious	N/A	74/2,649 (2.8%)	457/2,312 (19.8%)	RR 0.16 (0.13–0.20)	N/A	⊕⊕⊕O Moderate	Critical
Angina frequency	8	RCT	Serious	Serious	Not serious	Not serious	N/A	316	299	N/A	SMD 0.56 lower (0.91–0.21 lower)	⊕⊕OO Low	Critical
TC	12	RCT	Serious	Very serious	Not serious	Not serious	N/A	641	560	N/A	SMD 1.32 lower (1.88–0.76 lower)	⊕OOO Very low	Critical
TG	11	RCT	Serious	Very serious	Not serious	Not serious	N/A	581	509	N/A	SMD 0.78 lower (1.01–0.55 lower)	⊕OOO Very low	Critical
LDL-C	7	RCT	Serious	Very serious	Not serious	Not serious	N/A	436	403	N/A	MD 0.75 lower (1.07–0.42 lower)	⊕OOO Very low	Critical
FIB	8	RCT	Serious	Very serious	Not serious	Not serious	N/A	789	727	N/A	SMD 0.76 lower (1.05–0.48 lower)	⊕OOO Very low	Critical
Nitroglycerine consumption	4	RCT	Serious	Very serious	Not serious	Serious	N/A	135	125	N/A	SMD 0.67 lower (1.27–0.08 lower)	⊕OOO Very low	Critical

Abbreviations: CI, confidence interval; SMD, std mean difference.

## 4 Discussion

### 4.1 Summary of findings and clinical implications

This systematic review and meta-analysis synthesized evidence from 93 randomized controlled trials involving 10,208 patients with SAP. Overall, CDP as an adjunct to conventional therapy was associated with potential clinical benefits. Compared with conventional therapy alone, CDP significantly improved angina efficacy and electrocardiographic outcomes and reduced angina frequency and duration. Improvements were also reported in several secondary outcomes related to cardiac function, lipid profiles, inflammation, endothelial function, and coagulation. Compared with nitrates in combination with conventional therapy, CDP likewise showed favorable effects across multiple symptomatic, electrocardiographic, and physiological outcomes. However, substantial heterogeneity was observed for several outcomes, and the certainty of evidence ranged from moderate to very low. Therefore, these findings should be interpreted cautiously given the methodological limitations and uncertainty of the available evidence.

The pooled safety analyses suggested that CDP was generally well tolerated during the treatment periods evaluated. No significant difference in adverse events was observed between CDP plus conventional therapy and conventional therapy alone, whereas fewer adverse events were reported for CDP-containing regimens than for nitrate-containing regimens. Subgroup analyses showed favorable effects on several outcomes in both the ≤30-day and >30-day subgroups, with no significant subgroup differences for most outcomes. Therefore, the available evidence does not establish a differential effect according to treatment duration or an optimal treatment course for CDP.

### 4.2 Comparison with previous evidence and certainty of findings

Previous systematic reviews have reported favorable effects of CDP while consistently identifying methodological limitations in the primary trials. As direct comparisons between CDP and nitrates have already been evaluated, this comparison is not itself novel (Zhang et al., 2023). The present review updates the evidence within two predefined background-therapy frameworks and shows, using RoB 2 and GRADE, that concerns regarding risk of bias, heterogeneity, and evidence certainty persist despite the expanded evidence base (Supplementary Appendix SV).

Substantial heterogeneity was observed across multiple outcomes, particularly those related to angina symptoms, lipid parameters, and biomarkers. Treatment duration did not adequately explain this heterogeneity, as no significant subgroup differences were identified for most outcomes. Differences in patient characteristics, disease severity, background therapies, outcome definitions and measurement methods, treatment regimens, and study quality may have contributed to the observed variability, although these potential sources could not be adequately explored because of incomplete reporting.

Methodological limitations were also common. Many trials provided insufficient information regarding randomization and allocation concealment, lacked prospective registration or predefined protocols, and rarely implemented blinding despite the subjective nature of several outcomes. These limitations may have introduced bias and reduced confidence in the pooled estimates. In particular, the lack of prospective protocols or trial registration prevented complete assessment of selective outcome reporting. These challenges are consistent with broader concerns regarding the design and reporting of clinical trials involving traditional medicine interventions (Zhang et al., 2025).

Accordingly, the GRADE assessment showed that the certainty of evidence ranged from moderate to very low, with downgrading primarily due to risk of bias, inconsistency, and, for some outcomes, imprecision. Although sensitivity analyses generally did not materially alter the pooled estimates, statistical stability does not overcome limitations in the methodological quality of the primary studies. The trials remained eligible for quantitative synthesis, but the pooled estimates should be interpreted as evidence of potential treatment effects rather than definitive evidence of efficacy.

### 4.3 Preparation characteristics and potential mechanisms

The ConPhyMP assessment identified substantial deficiencies in preparation-specific reporting. None of the 93 RCTs adequately reported botanical authentication or extraction and processing procedures, only one sufficiently described the finished commercial product, and none provided chemical fingerprinting or comparison with official reference standards meeting the ConPhyMP criteria. Consequently, the comparability and reproducibility of the CDP preparations across trials could not be independently verified, limiting confidence in the pooled estimates.

The potential clinical benefits of CDP may be related to its multitarget pharmacological effects. CDP consists of *S. miltiorrhiza* Bunge, P. notoginseng (Burkill) F.H.Chen, and Borneolum Syntheticum and contains multiple bioactive metabolites that may modulate endothelial dysfunction, inflammation, oxidative stress, thrombosis, and metabolic disorders. Emerging evidence suggests that traditional medicine interventions may regulate ischemic cardiovascular injury through complex molecular mechanisms, including gene expression and post-transcriptional regulation (Sun et al., 2026).

Endothelial dysfunction and impaired coronary microcirculation contribute to myocardial ischemia in SAP. *Salvia miltiorrhiza* Bunge and its metabolites, including salvianolic acids and tanshinones, may enhance NO production and reduce oxidative injury (Cheng et al., 2021). Consistently, the included studies reported increased NO and decreased ET-1 following CDP treatment. CDP and its bioactive metabolites may also attenuate inflammation and oxidative stress (Fu et al., 2023), which are involved in coronary atherosclerosis and myocardial ischemic injury (Engelen et al., 2022; Roy et al., 2022). The reported reductions in CRP and increases in SOD are consistent with these potential mechanisms. Nevertheless, these findings provide biological plausibility rather than direct evidence of clinical efficacy.

### 4.4 Safety, strengths, and limitations

Safety data were reported in only 56 of the 93 RCTs. Adverse events were mainly mild, including headache, dizziness, gastrointestinal symptoms, hypotension, palpitations, and flushing. Although no serious treatment-related events were reported, incomplete reporting and short follow-up preclude conclusions about rare or long-term risks. Potential interactions with concomitant cardiovascular medications were also inadequately assessed; therefore, the available evidence supports only short-term tolerability rather than established long-term safety.

This study synthesized a large and updated evidence base of 93 randomized controlled trials involving more than 10,000 patients with SAP. The predefined therapeutic comparisons, subgroup and sensitivity analyses, and GRADE assessment enabled comprehensive evaluation of the available evidence. The incorporation of the ConPhyMP framework additionally allowed systematic assessment of preparation-specific reporting and characterization.

Several limitations should be acknowledged. Most studies were conducted in China and published in Chinese-language journals, potentially limiting generalizability and increasing the risk of language and publication bias. Methodological limitations, including inadequate reporting of randomization, allocation concealment and blinding, and the absence of prospective registration, reduced confidence in the pooled estimates. Substantial heterogeneity persisted for several outcomes, and incomplete preparation-specific reporting limited assessment of comparability across trials. Finally, relatively short treatment and follow-up periods, limited safety and interaction monitoring, and reliance on surrogate or symptom-based outcomes restrict conclusions regarding long-term safety and clinically important outcomes such as major adverse cardiovascular events and cardiovascular mortality.

### 4.5 Future perspectives

Future research should prioritize well-designed, multicenter, double-blind trials with adequate randomization and allocation concealment, prospective registration, standardized outcome definitions, and longer follow-up. Clinically meaningful endpoints, including major adverse cardiovascular events, myocardial infarction, cardiovascular mortality, and hospitalization, should be prioritized over reliance on surrogate outcomes. These requirements are consistent with international efforts to improve the methodological rigor and transparency of clinical trials involving traditional medicine (Zhang et al., 2025).

Future trials should also improve preparation-specific reporting in accordance with established standards such as the ConPhyMP framework, including botanical identity, manufacturing procedures, product specifications, quality control, and phytochemical characterization. Adverse events and potential interactions with concomitant cardiovascular medications should be systematically monitored. Validated quality-of-life measures should also be incorporated to assess patient-centered benefits. Integration of pharmacological, mechanistic, and clinical evidence may further clarify the relationship between the biological effects of CDP and clinically meaningful outcomes.

## 5 Conclusion

In conclusion, current evidence suggests that CDP, as an adjunct to conventional therapy, may provide symptomatic and functional benefits in patients with stable angina pectoris. However, given the limited certainty of evidence, well-designed randomized controlled trials with standardized reporting of CDP preparation, longer follow-up, and clinically relevant outcomes are needed to confirm these findings.

## Data Availability

The original contributions presented in the study are included in the article/Supplementary Material, further inquiries can be directed to the corresponding authors.

## Appendix A

The accepted scientific names and families were verified using Plants of the World Online (POWO), and the botanical drug names were obtained from the Chinese Pharmacopoeia. (Plants of the World Online, 2026).

**Table:**

Accepted scientific name	Family	Pharmacopoeial botanical drug name	POWO status
*Salvia miltiorrhiza* Bunge	Lamiaceae	Salviae miltiorrhizae radix et rhizoma	Accepted
*Panax notoginseng* (Burkill) F.H.Chen	Araliaceae	Notoginseng radix et rhizoma	Accepted

Synthetic borneol (Borneolum Syntheticum) is not a botanical species and therefore does not require taxonomic validation.
