# Supplementary material for: Efficacy and safety of Compound Danshen Dripping Pills as an adjunct to conventional therapy for stable angina pectoris: an updated systematic review and meta-analysis of randomized controlled trials
Source: Front Pharmacol. 2026 Sep 24;17:1952286. doi: 10.3389/fphar.2026.1952286 (PMC13645763; doi:10.3389/fphar.2026.1952286)
Supplement: Supplementary file 1 [file Supplementaryfile1.docx]

***Supplementary Appendix SⅠ***

**CNKI**:

(SU = '稳定型心绞痛' OR SU = '稳定性心绞痛' OR SU = '慢性稳定性心绞痛' OR SU = '稳定心绞痛' OR SU = '稳定心绞疼' OR SU = '稳定心绞病' OR SU = '劳力性心绞痛' OR SU = '劳力型心绞痛' OR SU = '劳力心绞痛' OR SU = '劳累心绞痛' OR SU = '冠心病心绞痛' OR SU = '心绞痛' OR SU = '稳定性冠心病' OR SU = '慢性冠脉综合征' OR SU = '慢性冠脉综合症' OR SU = '慢性冠状动脉综合征' OR SU = '慢性冠状动脉综合症' OR SU = '胸痹' OR SU = '心痛') AND (SU = '复方丹参滴丸' OR SU = '复方丹参丸' OR SU = '复方丹参制剂') AND (SU = '随机' OR SU = '临床' OR SU = '临床试验' OR SU = '临床研究' OR SU = 'RCT' OR SU = '疗效' OR SU = '有效性' OR SU = '安全性')

**VIP**:

M = (稳定型心绞痛 OR 稳定性心绞痛 OR 慢性稳定性心绞痛 OR 稳定心绞痛 OR 稳定心绞疼 OR 稳定心绞病 OR 劳力性心绞痛 OR 劳力型心绞痛 OR 劳力心绞痛 OR 劳累心绞痛 OR 冠心病心绞痛 OR 心绞痛 OR 稳定性冠心病 OR 慢性冠脉综合征 OR 慢性冠脉综合症 OR 慢性冠状动脉综合征 OR 慢性冠状动脉综合症 OR 胸痹 OR 心痛) AND M = (复方丹参滴丸 OR 复方丹参丸 OR 复方丹参制剂) AND U = (随机 OR 临床 OR 临床试验 OR 临床研究 OR RCT OR 疗效 OR 有效性 OR 安全性)

**Wanfang**:

主题:(稳定型心绞痛 OR 稳定性心绞痛 OR 慢性稳定性心绞痛 OR 稳定心绞痛 OR 稳定心绞疼 OR 稳定心绞病 OR 劳力性心绞痛 OR 劳力型心绞痛 OR 劳力心绞痛 OR 劳累心绞痛 OR 冠心病心绞痛 OR 心绞痛 OR 稳定性冠心病 OR 慢性冠脉综合征 OR 慢性冠脉综合症 OR 慢性冠状动脉综合征 OR 慢性冠状动脉综合症 OR 胸痹 OR 心痛) AND 主题:(复方丹参滴丸 OR 复方丹参丸 OR 复方丹参制剂) AND 全部:(随机 OR 临床 OR 临床试验 OR 临床研究 OR RCT OR 疗效 OR 有效性 OR 安全性)

**SinoMed**:

("随机"[全部字段:智能] OR "临床"[常用字段:智能] OR "临床试验"[常用字段:智能] OR "临床研究"[常用字段:智能] OR "RCT"[常用字段:智能] OR "疗效"[常用字段:智能] OR "有效性"[常用字段:智能] OR "安全性"[常用字段:智能]) AND ("复方丹参滴丸"[常用字段:智能] OR "复方丹参丸"[常用字段:智能] OR "复方丹参制剂"[常用字段:智能]) AND ("稳定型心绞痛"[常用字段:智能] OR "稳定性心绞痛"[常用字段:智能] OR "慢性稳定性心绞痛"[常用字段:智能] OR "稳定心绞痛"[常用字段:智能] OR "稳定心绞疼"[常用字段:智能] OR "稳定心绞病"[常用字段:智能] OR "劳力性心绞痛"[常用字段:智能] OR "劳力型心绞痛"[常用字段:智能] OR "劳累心绞痛"[常用字段:智能] OR "冠心病心绞痛"[常用字段:智能] OR "心绞痛"[常用字段:智能] OR "稳定性冠心病"[常用字段:智能] OR "慢性冠脉综合征"[常用字段:智能] OR "慢性冠脉综合症"[常用字段:智能] OR "胸痹"[常用字段:智能] OR "心痛"[常用字段:智能])

**Web of Science**:

#1 TS=("Angina, Stable" OR "Anginas, Stable" OR "Stable Angina" OR "Stable Anginas" OR "Angina Pectoris, Stable" OR "Angina Pectori, Stable" OR "Pectoris, Stable Angina" OR "Pectori, Stable Angina" OR "Stable Angina Pectori" OR "Stable Angina Pectoris" OR "Chronic Stable Angina" OR "Angina, Chronic Stable" OR "Anginas, Chronic Stable" OR "Chronic Stable Anginas" OR "Stable Angina, Chronic" OR "Stable Anginas, Chronic" OR "Angina Pectoris" OR "Angor Pectoris" OR "Stenocardia" OR "Stenocardias" OR "Stable Coronary Artery Disease" OR "Chronic Coronary Syndromes")
#2 TS=("Compound Danshen Dripping Pill*" OR "Compound Danshen Pill*" OR "Compound Danshen Preparation*" OR "Guanxin Danshen Dripping Pill*" OR "Fufang Danshen" OR "Fufang Danshen Dripping Pill*" OR "Fufang Danshen Diwan" OR "Fufang Danshen Pill*" OR "Fufang Danshen Preparation*")
#3 ALL=("randomized controlled trial" OR "randomized clinical trial" OR "randomized trial" OR "clinical trial")
#4 = #1 AND #2 AND #3

**Pubmed**:

#1 "Angina, Stable" [MeSH Terms] OR "Anginas, Stable" [Title/Abstract] OR "Stable Angina" [Title/Abstract] OR "Stable Anginas" [Title/Abstract] OR "Angina Pectoris, Stable" [Title/Abstract] OR "Angina Pectori, Stable" [Title/Abstract] OR "Pectoris, Stable Angina" [Title/Abstract] OR "Pectori, Stable Angina" [Title/Abstract] OR "Stable Angina Pectori" [Title/Abstract] OR "Stable Angina Pectoris" [Title/Abstract] OR "Chronic Stable Angina" [Title/Abstract] OR "Angina, Chronic Stable" [Title/Abstract] OR "Anginas, Chronic Stable" [Title/Abstract] OR "Chronic Stable Anginas" [Title/Abstract] OR "Stable Angina, Chronic" [Title/Abstract] OR "Stable Anginas, Chronic" [Title/Abstract] OR "Angina Pectoris"[MeSH Terms] OR "Angor Pectoris"[Title/Abstract] OR "Stenocardia" [Title/Abstract] OR "Stenocardias"[Title/Abstract] OR "Stable Coronary Artery Disease" [Title/Abstract] OR "Chronic Coronary Syndromes" [Title/Abstract]

#2 "Compound Danshen Dripping Pill" [Title/Abstract] OR "Compound Danshen Pill" [Title/Abstract] OR "Compound Danshen Preparation" [Title/Abstract] OR "Guanxin Danshen Dripping Pill" [Title/Abstract] OR "Fufang Danshen" [Title/Abstract] OR "Fufang Danshen Dripping Pill" [Title/Abstract] OR "Fufang Danshen Diwan" [Title/Abstract] OR "Fufang Danshen Pill" [Title/Abstract] OR "Fufang Danshen Preparation" [Title/Abstract]

#3 "randomized controlled trial" [Publication type] OR "randomized clinical trial" [Publication type] OR "randomized trial" [Publication type] OR "clinical trial" [Publication type] OR "randomized controlled trial" [Title/Abstract] OR "randomized clinical trial" [Title/Abstract] OR "randomized trial" [Title/Abstract] OR "clinical trial" [Title/Abstract]

#4 #1 AND #2 AND #3

**Embase**:

#1 'Angina, Stable':ti,ab,kw OR 'Anginas, Stable':ti,ab,kw OR 'Stable Angina':ti,ab,kw OR 'Stable Anginas':ti,ab,kw OR 'Angina Pectoris, Stable':ti,ab,kw OR 'Angina Pectori, Stable':ti,ab,kw OR 'Pectoris, Stable Angina':ti,ab,kw OR 'Pectori, Stable Angina':ti,ab,kw OR 'Stable Angina Pectori':ti,ab,kw OR 'Stable Angina Pectoris':ti,ab,kw OR 'Chronic Stable Angina':ti,ab,kw OR 'Angina, Chronic Stable':ti,ab,kw OR 'Anginas, Chronic Stable':ti,ab,kw OR 'Chronic Stable Anginas':ti,ab,kw OR 'Stable Angina, Chronic':ti,ab,kw OR 'Stable Anginas, Chronic':ti,ab,kw OR 'Angina Pectoris':ti,ab,kw OR 'Angor Pectoris':ti,ab,kw OR 'Stenocardia':ti,ab,kw OR 'Stenocardias':ti,ab,kw OR 'Stable Coronary Artery Disease':ti,ab,kw OR 'Chronic Coronary Syndromes':ti,ab,kw

#2 'Compound Danshen Dripping Pill'/exp OR 'Compound Danshen Pill':ti,ab,kw OR 'Compound Danshen Preparation':ti,ab,kw OR 'Guanxin Danshen Dripping Pill':ti,ab,kw OR 'Fufang Danshen':ti,ab,kw OR 'Fufang Danshen Dripping Pill':ti,ab,kw OR 'Fufang Danshen Diwan':ti,ab,kw OR 'Fufang Danshen Pill':ti,ab,kw OR 'Fufang Danshen Preparation':ti,ab,kw

#3 'randomized controlled trial'/exp OR 'randomized controlled trial':ti,ab,kw OR 'randomized clinical trial':ti,ab,kw OR 'randomized trial':ti,ab,kw OR 'clinical trial':ti,ab,kw

#4 = #1 AND #2 AND #3

**The Cochrane Library**:

#1 MeSH descriptor: [Angina, Stable] explode all trees
#2 (Angina, Stable OR Anginas, Stable OR Stable Angina OR Stable Anginas OR Angina Pectoris, Stable OR Angina Pectori, Stable OR Pectoris, Stable Angina OR Pectori, Stable Angina OR Stable Angina Pectori OR Stable Angina Pectoris OR Chronic Stable Angina OR Angina, Chronic Stable OR Anginas, Chronic Stable OR Chronic Stable Anginas OR Stable Angina, Chronic OR Stable Anginas, Chronic OR Angina Pectoris OR Angor Pectoris OR Stenocardia OR Stenocardias OR Stable Coronary Artery Disease OR Chronic Coronary Syndromes):ti,ab,kw
#3 = #1 OR #2
#4 MeSH descriptor: [Compound Danshen Dripping Pill] explode all trees
#5 (Compound Danshen Dripping Pill OR Compound Danshen Pill OR Compound Danshen Preparation OR Guanxin Danshen Dripping Pill OR Fufang Danshen OR Fufang Danshen Dripping Pill OR Fufang Danshen Diwan OR Fufang Danshen Pill OR Fufang Danshen Preparation):ti,ab,kw
#6 = #4 OR #5
#7 MeSH descriptor: [Randomized Controlled Trial] explode all trees
#8 (randomized controlled trial OR randomized clinical trial OR randomized trial OR clinical trial):ti,ab,kw
#9 = #7 OR #8
#10 = #3 AND #6 AND #9
