# Supplementary material for: Efficacy and safety of Compound Danshen Dripping Pills as an adjunct to conventional therapy for stable angina pectoris: an updated systematic review and meta-analysis of randomized controlled trials
Source: Front Pharmacol. 2026 Sep 24;17:1952286. doi: 10.3389/fphar.2026.1952286 (PMC13645763; doi:10.3389/fphar.2026.1952286)
Supplement: Supplementary file 2 [file Supplementaryfile2.docx]

***Supplementary Appendix SⅡ：Sensitivity analyses***

1. **CDP+CT vs. CT**
   1.1 **Angina efficacy**

1.2 **ECG efficacy**
    1.2.1 **Whole group analysis**

1.2.2 **≤ 30 days**

1.2.3 **＞ 30 days**

1.3 **Adverse events**

1.4 **Angina frequency**
    1.4.1 **Whole group analysis**

1.4.2 **≤ 30 days**

1.4.3 **＞ 30 days**

1.5 **Angina duration**
    1.5.1 **Whole group analysis**

1.5.2 **≤ 30 days**

1.5.3 **＞ 30 days**

1.6 **LVEF**
    1.6.1 **Whole group analysis**

1.6.2 **≤ 30 days**

2. **CDP+CT vs. Nitrates+CT**
   2.1 **Angina efficacy**
       2.1.1 **Whole group analysis**

2.1.2 **≤ 30 days**

2.1.3 **＞ 30 days**

2.2 **ECG efficacy**

2.3 **Adverse events**

2.4 **Angina frequency**
    2.4.1 **Whole group analysis**

2.4.2 **＞ 30 days**

2.5 **Total cholesterol**
    2.5.1 **Whole group analysis**

2.5.2 **≤ 30 days**

2.5.3 **＞ 30 days**

2.6 **Triglyceride**
    2.6.1 **Whole group analysis**

2.6.2 **≤ 30 days**

2.6.3 **＞ 30 days**

2.7 **Low-density lipoprotein cholesterol**
    2.7.1 **Whole group analysis**

2.7.2 **≤ 30 days**

2.7.3 **＞ 30 days**

2.8 **Fibrinogen**
    2.8.1 **Whole group analysis**

2.8.2 **≤ 30 days**

2.8.3 **＞ 30 days**

2.9 **Nitroglycerine consumption**
    2.9.1 **Whole group analysis**

2.9.2 **＞ 30 days**
