# Supplementary material for: Efficacy and safety of Compound Danshen Dripping Pills as an adjunct to conventional therapy for stable angina pectoris: an updated systematic review and meta-analysis of randomized controlled trials
Source: Front Pharmacol. 2026 Sep 24;17:1952286. doi: 10.3389/fphar.2026.1952286 (PMC13645763; doi:10.3389/fphar.2026.1952286)
Supplement: Supplementary file 3 [file Supplementaryfile3.docx]

**Classification of Reported Adverse Events**

| **Standardized adverse-event category** | **Included events** | **CDP + CT, n** | **CT, n** | **Total, n** |
| --- | --- | --- | --- | --- |
| **CDP+CT vs. CT** | | | | |
| Neurological symptoms | Headache, dizziness, vertigo, head distension, drowsiness, and combined headache/dizziness | 24 | 32 | 56 |
| Gastrointestinal symptoms | Gastrointestinal reactions or discomfort, nausea, vomiting, abdominal pain, and diarrhea | 13 | 13 | 26 |
| Cardiovascular and vasomotor symptoms | Flushing, hypotension, increased heart rate, and dizziness/palpitations | 12 | 12 | 24 |
| General symptoms | Fatigue and dry mouth | 4 | 6 | 10 |
| Laboratory abnormalities | Abnormal liver/renal function and abnormal routine blood tests | 2 | 2 | 4 |
| Total | **—** | 55 | 65 | 120 |
| **CDP + CT vs. Nitrates + CT** | | | | |
| Neurological and vasomotor symptoms | Headache, dizziness, head distension, flushing/burning sensation, palpitations, orthostatic hypotension, fever, and mixed symptom clusters | 7 | 527 | 534 |
| Gastrointestinal symptoms | Gastric or abdominal discomfort, gastrointestinal reactions, acid regurgitation, nausea, and vomiting | 63 | 33 | 96 |
| Laboratory abnormalities | Abnormal liver function | 4 | 2 | 6 |
| Unspecified adverse reactions | Adverse reactions not otherwise specified | 1 | 0 | 1 |
| Total | **—** | 75 | 562 | 637 |
