# Supplementary material for: Efficacy and safety of Compound Danshen Dripping Pills as an adjunct to conventional therapy for stable angina pectoris: an updated systematic review and meta-analysis of randomized controlled trials
Source: Front Pharmacol. 2026 Sep 24;17:1952286. doi: 10.3389/fphar.2026.1952286 (PMC13645763; doi:10.3389/fphar.2026.1952286)
Supplement: Supplementary file 4 [file Table1.docx]

| **Study** | **Population** | **Search period/date** | **Included studies/participants** | **Study design** | **Intervention/comparator** | **Main outcomes** | **Subgroup/sensitivity analysis** | **Risk-of-bias assessment** | **GRADE** | **Safety assessment** | **Preparation-specific characterization** | **ConPhyMP/equivalent** | **Key limitations/remaining gaps** |
| --- | --- | --- | --- | --- | --- | --- | --- | --- | --- | --- | --- | --- | --- |
| Li et al. (2011) | Stable angina pectoris | MEDLINE and CNKI; studies published 1966–2010 | 15 RCTs; 1,536 participants | RCTs | DSP vs. isosorbide dinitrate; DSP + routine therapy vs. isosorbide dinitrate + routine therapy | Angina symptoms; ECG efficacy | NR | Basic methodological assessment reported | No | Limited | No systematic preparation-specific assessment identified | No | Restricted databases/outcomes; no formal certainty-of-evidence assessment; no systematic evaluation of preparation comparability |
| Ng et al. (2012) | Angina pectoris | Studies published 1994–2011; last search Apr 7, 2011 | 9 RCTs; 926 participants | RCTs | DSP vs. Di'ao Xinxuekang | Overall clinical efficacy; ECG efficacy | Yes; subgroup and sensitivity analyses | Jadad scale + Cochrane risk-of-bias tool | No | Not a major focus | No systematic preparation-specific assessment identified | No | Small evidence base; limited outcomes; no GRADE or preparation-comparability assessment |
| Jia et al. (2012) | Angina pectoris | RCTs published 1994–2009 | 60 RCTs; 6,931 participants | RCTs | DSP vs. isosorbide dinitrate | Symptom improvement; ECG improvement | Yes; subgroup and sensitivity analyses | Refined Jadad scale | No | Limited | Dose and treatment duration considered; no systematic phytochemical/preparation characterization | No | Outcomes largely restricted to symptom and ECG response; methodological quality of primary trials remained limited |
| Ye et al. (2013) | Coronary heart disease with angina pectoris | Databases searched from inception; exact final date NR | NR from available report | Controlled clinical studies in systematic review/meta-analysis | CDP-related treatment vs. control therapies | Angina attacks; ECG; TC; TG; heart-rate variability; safety | Some quantitative/descriptive synthesis | Cochrane-based quality assessment | No | Yes | No systematic preparation-specific assessment identified | No | Intervention characterization and certainty of evidence not systematically addressed |
| Yao et al. (2015) | Angina pectoris | Updated review after earlier DSP–ISDN syntheses | Updated RCT evidence; exact total NR in available record | RCTs | CDP vs. isosorbide dinitrate | Clinical efficacy; ECG efficacy | Sensitivity analysis | Based on previous systematic-review framework | No | Limited | No systematic preparation-specific assessment identified | No | Primarily an update of an established comparison; restricted outcome scope; no GRADE/preparation assessment |
| Jia and Leung (2017) | Angina pectoris | RCTs published 1994–2016 | 109 RCTs; 11,973 participants | RCTs | DSP vs. isosorbide dinitrate | Symptomatic improvement; ECG improvement | Yes; subgroup, sensitivity, meta-regression, publication-bias analyses | Cochrane risk-of-bias tool | Yes | Not a principal outcome | No systematic preparation-specific characterization identified | No | High risk of bias in randomization/blinding and heterogeneity; overall GRADE certainty remained low |
| Qiang et al. (2020) | Coronary heart disease with angina pectoris | CNKI, VIP, CBM, Wanfang, PubMed and Cochrane Library; published 2020 | 39 studies; 3,941 participants | Predominantly randomized comparative studies | CDP + conventional chemical therapy vs. conventional therapy | Clinical/ECG efficacy; angina frequency/duration; hs-CRP; ET; platelet aggregation; GMP-140; FIB; adverse reactions | Yes; subgroup and publication-bias analyses | Cochrane Handbook-based quality assessment | No | Yes; 15 studies reported adverse reactions | No systematic preparation-specific assessment identified | No | Primary-study quality described as low; no formal GRADE; preparation identity/comparability not systematically examined |
| Zhang et al. (2023) | Stable angina pectoris | Databases from inception to April 2023 | 29 RCTs; 3,185 participants | RCTs | CDDP vs. nitrates | Symptom efficacy; ECG efficacy; adverse drug reactions | Yes; duration-based subgroup analyses; sensitivity analyses | Cochrane risk-of-bias approach | Yes | Yes | No ConPhyMP-based or systematic phytochemical/preparation characterization identified | No | Random sequence generation, allocation concealment and blinding inadequately reported; reporting bias unclear; certainty very low to low |
| Wang et al. (2024) | Angina pectoris | Databases from inception to November 2022 | 59 RCTs + 11 cohort studies; 7,245 participants | RCTs + cohort studies | CDDP + isosorbide mononitrate vs. isosorbide mononitrate alone | Angina/ECG efficacy; TC; TG; angina frequency/duration; adverse events | Yes; sensitivity and publication-bias analyses | Study-design-specific methodological assessment | No formal GRADE identified | Yes | No ConPhyMP-based systematic preparation characterization identified | No | Included observational studies; restricted to ISMN-based therapy; no formal certainty-of-evidence or preparation-comparability assessment |
| Liu and Zhu (2025) | Stable angina pectoris | CNKI and PubMed from inception to July 31, 2024 | 10 RCTs | RCT-focused perspective/systematic clinical evidence review | CDDP monotherapy or combination therapy | Clinical efficacy; safety; pharmacological mechanisms | Not a conventional quantitative meta-analysis | Narrative methodological appraisal | No formal GRADE synthesis identified | Yes, descriptively | No systematic ConPhyMP assessment identified | No | Limited database coverage and selected evidence base; largely narrative synthesis; no quantitative preparation-comparability assessment |
| Present review | Stable angina pectoris | Search updated to final prespecified date in revised manuscript | 93 RCTs; >10,000 participants | RCTs only | CDP + CT vs. CT; CDP + CT vs. nitrates + matched CT | Angina/ECG efficacy; angina frequency/duration; nitroglycerin consumption; TC; TG; LDL-C; FIB; other outcomes; adverse events | Yes; treatment-duration subgroup analyses and leave-one-out sensitivity analyses | Cochrane Risk of Bias tool for randomized trials (RoB 2) | Yes | Yes; study-level adverse-event characterization | Yes; composition, botanical identity, preparation/process reporting, product characteristics and analytical characterization assessed | Yes; ConPhyMP | Expanded evidence base but persistent RoB, heterogeneity and reporting limitations; ConPhyMP identifies limitations in preparation-specific reporting and cross-trial comparability |

**Appendix Table A1. Characteristics and methodological features of previous systematic reviews and meta-analyses evaluating Compound Danshen Dripping Pills for angina pectoris**

**Abbreviations:** CDP/CDDP/DSP, Compound Danshen Dripping Pills/Danshen Dripping Pills as termed in the original reviews; SAP, stable angina pectoris; CT, conventional therapy; ISDN, isosorbide dinitrate; ISMN, isosorbide mononitrate; ECG, electrocardiogram; TC, total cholesterol; TG, triglycerides; LDL-C, low-density lipoprotein cholesterol; FIB, fibrinogen; ET, endothelin; hs-CRP, high-sensitivity C-reactive protein; AE, adverse event; ADR, adverse drug reaction; RoB, risk of bias; GRADE, Grading of Recommendations Assessment, Development and Evaluation; NR, not reported or not reliably ascertainable from the available report; ConPhyMP, Consensus statement on the Phytochemical Characterisation of Medicinal Plant extracts.
